# Prenatal Diagnosis of Adrenal Neuroblastoma: A Case Report with a Brief Review of the Literature

**DOI:** 10.1155/2013/506490

**Published:** 2013-03-27

**Authors:** Onur Erol, Dinç Süren, Melek Büyükkınacı Erol

**Affiliations:** ^1^Department of Obstetrics and Gynecology, Antalya Education and Research Hospital, 07030 Antalya, Turkey; ^2^Department of Pathology, Antalya Education and Research Hospital, 07030 Antalya, Turkey; ^3^Department of Obstetrics and Gynecology, Medstar Hospital, 07030 Antalya, Turkey

## Abstract

A case of adrenal cystic neuroblastoma detected at 37 weeks of gestation is reported. Postnatal ultrasonographic examination showed slightly increased in size demonstrating marked septations within the cyst. After the tumor was resected, histopathological examinations confirmed the diagnosis. The patient is developing normally at 1 year of age.

## 1. Introduction

Neuroblastoma is the most common solid tumor in children under 1 year of age [1]. The overall incidence is 58/1,000,000 infants per year [2]. This tumor is derived from neural crest cells, and it can arise anywhere in the sympathetic nervous system. Environmental factors or parental exposures that significantly impact on disease occurrences have been identified; also in 1-2% of cases there is a family history [3]. Specific tumor suppressor gene responsible for genetic abnormality in neuroblastoma has not been identified to date [4].

Prenatal diagnosis of neuroblastoma was first reported by Fénart et al. [5]. Neuroblastoma is normally diagnosed during the third trimester, and adrenal location is the most observed origin with a ninety percent. The prognosis depends on the extent of disease at diagnosis. Follow-up ultrasonographic examinations should be performed every two weeks from diagnosis to term in order to monitor the size of the mass and to detect antenatal metastatic complications [6].

We have reported a case of cystic adrenal neuroblastoma that was growing rapidly postnatally with a brief review of the literature.

## 2. Case Presentation

**Figure 1:**
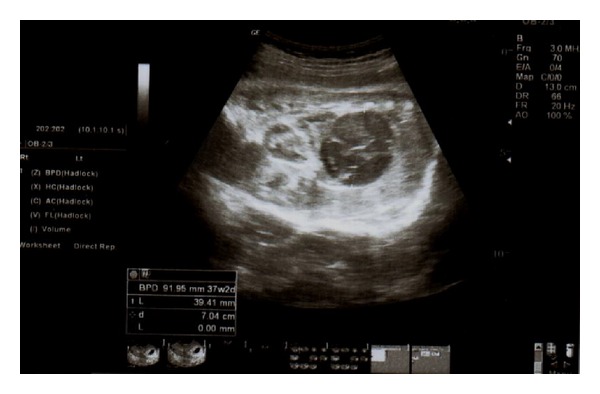
Ultrasonographic appearance of cystic neuroblastoma at 37 weeks of gestation.

**Figure 2:**
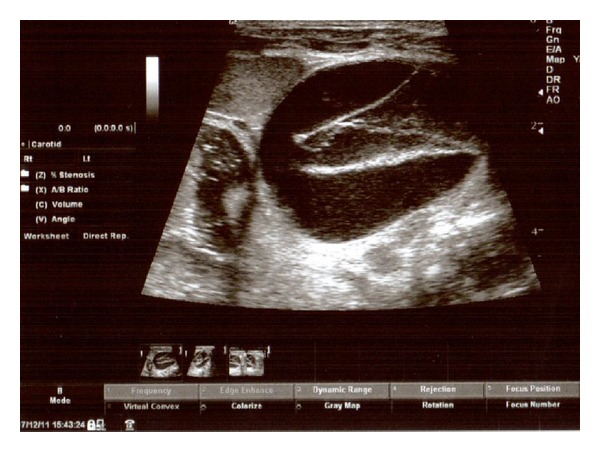
Postnatal ultrasonographic appearance with marked septations.

**Figure 3:**
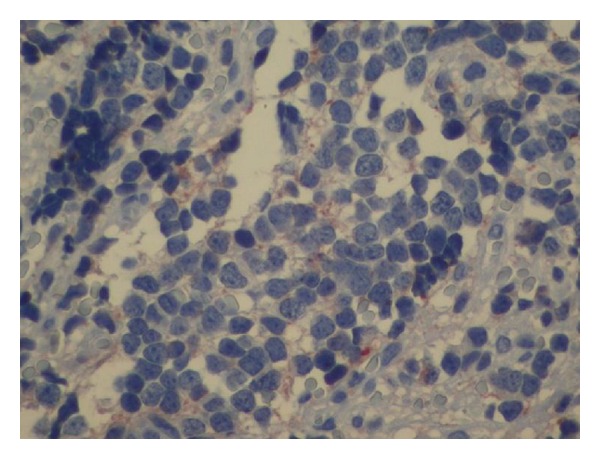
Histopathology of the cyst composed of small round cells with rosette formation (synaptophysin stain, 200x).

A 38-year-old woman, gravida 2 para 1, was referred to our center for assessment of cystic mass in fetal abdomen at 37 weeks of gestation. Her medical history was unremarkable. The detailed ultrasonographic evaluation revealed suprarenal cystic mass on the left side with a diameter of 45∗45 mm (Figure 1). The cyst was homogenous with intracystic septations suggesting intracystic hemorrhage. Color Doppler imaging of the mass revealed peripheral vascularization, and no blood flow was seen in the cyst. The spleen and left kidney were normal in appearance. The possibility of neuroblastoma was raised. No other fetal structural abnormalities were observed. Maternal urine homovanillic acid and vanilmandelic acid levels were normal. Followup sonography two weeks later demonstrated the same size of cyst with an 8/8 score on the fetal biophysical profile. The infant was then delivered by cesarean section due to fetal malpositioning (breech presentation) at 39 weeks 4 days of gestation. A male infant weighing 3100 gram was born, with Apgar scores of 8 at 1 minute and 10 at 5 minutes. Postnatal sonographic examination confirmed the presence of an adrenal tumor measuring 48∗50 mm in size. One week later cyst size was increased to 65∗70 mm (Figure 2). Laparotomy was performed on the 8 day postpartum, and the tumor was resected. Histopathological examination of the tumor was consistent with well-differentiated neuroblastoma with tumor-free margins, and within intracystic hemorrhage was noted. Immunohistochemical staining for chromogranin-A, vimentin, CD 99, and myogenin was negative with poor staining for synaptophysin (Figure 3). Tumor cytogenetic analysis showed no aneuploidy. The infant had an uncomplicated postoperative course and was discharged 10 days later. He required no further treatment and remained free of disease 1 year later.

## 3. Discussion

Improvements in prenatal imaging and widespread use of fetal ultrasonography have led to an increased rate of prenatal diagnosis of fetal neuroblastoma. The features of neuroblastoma on the antenatal ultrasound are variable and range from cystic, mixed solid and cystic, and completely solid with or without calcification. Patients with cystic neuroblastoma had a better outcome than those noncystic tumors [7]. The main differential diagnosis is adrenal haemorrhage which is the most common cause of adrenal mass during the perinatal period with an incidence of 1, 9/1000 live births [8]. It is important to differentiate benign adrenal lesions like adrenal hemorrhage or adrenal cysts from neuroblastoma. Magnetic resonance imaging (MRI) is the modality of choice for differentiation between these situations and to detect early metastatic complication of neuroblastoma in the perinatal period. Other suprarenal masses like extralobular pulmonary sequestration, adrenal abscess, adrenal nodular hyperplasia, adrenal cyst, bronchogenic cyst, and rarely adrenal carcinoma may mimic neuroblastoma, so differential diagnosis from these circumstances must be considered [9].

Reported prevalence of neuroblastoma cases in prenatally detected suprarenal masses is 81–85 % in the literature [10, 11]. The entity “neuroblastoma in situ” represents the collections of neuroblastoma cells, and it is important to distinguish this from clinically apparent neuroblastoma [12]. Cystic neuroblastoma is thought to be a form of neuroblastoma in situ that is associated with highest survival rate of all forms of neuroblastoma [13]. These tumors are characterized by Shimada favorable histology and biological markers such as aneuploid DNA content, lack of chromosome 1p deletion, and absence of MYCN (myelocytomatosis viral related oncogene) amplification [14, 15]. Conflicting correlation results between clinical course and biological markers are also stated in the literature [16, 17].

Relationship between neuroblastoma and congenital heart defects was emphasized in a report with a recommendation of echocardiography for congenital cardiovascular malformation screening in patients with newly diagnosed neuroblastoma [18]. Fetal hypertension and heart failure, invasion of erythropoietic tissue with tumor cells, and metastasis to the placenta may lead to development of fetal hydrops [19].

Urinary catecholamines are helpful in confirming the diagnosis of neuroblastoma; however, they will be normal in two-thirds of patients with prenatally diagnosed tumors, thus this test will not serve as a reliable diagnostic aid in every case [20]. Evaluation for the presence of metastatic disease must be considered with MRI, computerized tomography (CT) scanning, or *¹*²³I methyliodobenzylguanidine (MIBG) scintigraphy that is most sensitive for identifying metastatese in general [10]. Classification according to the International Neuroblastoma Staging System (INSS) is a combined clinical/surgical staging system that includes local extension of the tumor, lymph node involvement, extent of resection of the primary tumor, and presence of distant metastatic disease [21]. The liver was the main site of metastases followed in order by the bone marrow, skin, and lungs. Most prenatally diagnosed neuroblastoma is adrenal tumors at favorable stages (INSS stage I, II, or IV-S), and also rare region like pancreatic, cervical neuroblastoma have been reported [22, 23].

The treatment options for congenital neuroblastoma include surgery, chemotherapy followed by surgery or expectant management with ultrasonographic followup [24]. Most patients with stages 1 and 2 disease can be cured with surgery alone [15]. The use of chemotherapy is reserved for high stage metastatic disease. The strategy of surgical exploration of adrenal masses has been replaced by a wait and see strategy in recent years in low stage neuroblastoma [8]. Tumour regression in 11 of 12 cases of early stage neuroblastoma detected by mass screening was reported [25]. Dumbbell neuroblastoma is considered to be unresectable tumor, and preoperative chemotherapy is recommended [26]. The treatment is often followed by excision of the tumor.

## 4. Conclusion

Close sonographic monitoring of cystic adrenal masses during the first months life is helpful to detect early complications, and surgical excision could be performed safely during the neonatal period.
